# The Effect of Ginger (*Zingiber officinale* Roscoe) Aqueous Extract on Postprandial Glycemia in Nondiabetic Adults: A Randomized Controlled Trial

**DOI:** 10.3390/foods12051037

**Published:** 2023-03-01

**Authors:** Alda Diakos, Maria Leonor Silva, José Brito, Margarida Moncada, Maria Fernanda de Mesquita, Maria Alexandra Bernardo

**Affiliations:** Centro de Investigação Interdisciplinar Egas Moniz (CiiEM), Instituto Universitário Egas Moniz, Campus Universitário, Quinta da Granja, Monte de Caparica, 2829-511 Caparica, Portugal

**Keywords:** ginger, *Zingibre officinalle* Roscoe, postprandial glycemia, antioxidant, polyphenols, flavonoids

## Abstract

Ginger has shown beneficial effects on blood glucose control due to its antioxidant and anti-inflammatory properties. The present study investigated the effect of ginger aqueous extract on postprandial glucose levels in nondiabetic adults and characterized its antioxidant activity. Twenty-four nondiabetic participants were randomly assigned into two groups (NCT05152745), the intervention group (n = 12) and the control group (n = 12). Both groups were administered 200 mL of an oral glucose tolerance test (OGTT), after which participants in the intervention group ingested 100 mL of ginger extract (0.2 g/100 mL). Postprandial blood glucose was measured while fasting and after 30, 60, 90, and 120 min. The total phenolic content, flavonoid content, and antioxidant activity of ginger extract were quantified. In the intervention group, the incremental area under the curve for glucose levels decreased significantly (*p* < 0.001) and the maximum glucose concentration significantly reduced (*p* < 0.001). The extract possessed a polyphenolic content of 13.85 mg gallic acid equivalent/L, a flavonoid content of 3.35 mg quercetin equivalent/L, and a high superoxide radical inhibitory capacity (45.73%). This study showed that ginger has a beneficial effect on glucose homeostasis under acute conditions and encourages the use of ginger extract as a promising source of natural antioxidants.

## 1. Introduction

The postprandial blood glucose concentration has been reported as a key factor in glucose homeostasis control, which seems to be effective in preventing the development and progression of long-term diabetes complications [1]. According to epidemiological data, there is an association between cardiovascular and all-cause death and postprandial hyperglycemia status in nondiabetic patients [2]. In addition, the hyperglycemic status combined with clinical parameters can also predict an increased risk of developing diabetes [3].

It has been reported that postprandial glycemia profiles can be influenced by several factors, such as carbohydrate absorption, insulin and glucagon secretion and/or action, and glucose metabolism in different tissues [1]. Although the peak glucose concentration of nondiabetic individuals occurs about 60 min after the meal, the meal composition influences the magnitude and timing of the peak [1].

There is also evidence that, during hyperglycemic conditions, the oxygen free radicals are overproduced, leading to oxidative stress and cellular damage. This oxidative stress has been correlated with the development of diabetes complications [4]. 

Ginger (*Zingiber officinale* Roscoe) is a traditional herb belonging to Zingiberaceae family that has revealed beneficial effects on human health [5]. This herb has been used to treat nausea and vomiting, pain, metabolic syndrome, osteoarthritis, and obesity conditions [6,7,8,9,10]. In addition, it has been proposed that ginger possesses antioxidant and anti-inflammatory properties [11,12]. The main classes of the components responsible for ginger’s bioactivities include shogaols, gingerols, zingerone, and zingiberene [13,14]. It has been shown that these bioactive ginger compounds possess antidiabetic properties that are thought to enhance insulin secretion through the modulation of K_ATP_ channels [15]. In addition, 6-Gingerol potentiates the glucagon-like peptide 1 (GLP-1)-mediated glucose-stimulated insulin-secretion pathway in the pancreatic beta cell [16]. Another proposed mechanism of action postulates that the possible stimulation of Rab27a GTPase, in isolated islets, may contribute to the exocytosis of insulin-containing dense core granules. Increased Rab27a GTPase may also increase the translocation of the glucose transporter 4 (GLUT4) vesicle to the membrane of skeletal myocytes [16].

Currently, there is promising evidence of the beneficial properties of ginger extract, which seems to be effective in lowering blood glucose levels [17]. According to the Zhu et al. study, ethanolic ginger extract (200 mg/Kg body weight) demonstrated a significant antihyperglycemic effect in streptozotocin (STZ)—diabetic rats—for 20 days [17]. Ginger aqueous extract (500 mg/Kg body weight) significantly reduced blood glucose level after ginger treatment on the 8th day compared with the baseline in alloxan-induced diabetic rats [18]. However, recently published data on human studies have shown conflicting results regarding blood glucose control [19]. In Karimi et al.’s study, the ingestion of a ginger supplement (four capsules) (3 g/day) for 7 weeks did not significantly change blood glucose in the ginger group (6.5 ± 0.4 mmol/L) compared to the placebo group (6.5 ± 1 mmol/L) [20]. Additionally, in another study, the ingestion of a ginger capsule (1000 mg per day) for 10 weeks significantly reduced the fasting blood glucose by up to 20% in the nondiabetic adult ginger group at the end of the experimental protocol [21]. Conversely, in the Bordia et al. study, the ingestion of 5 g ginger powder (4 g per day) in nondiabetic patients for 3 months did not affect fasting and postprandial blood glucose levels [22].

Among the studies found in the literature focusing on the effect of ginger on blood sugar, few works have been developed on the effect of this herb on postprandial glycemia. Hung and co-workers (2022) demonstrated that a spice mix meal containing ginger significantly reduced postprandial glucose levels in obese and overweight adults [23]. In accordance with the lack of literature concerning ginger’s effect on the glucose response, the main aim of the present study was to investigate the effect of ginger (*Zingibre officinalle* Roscoe) aqueous extract (0.2 g/100 mL) on postprandial glucose levels in nondiabetic adults. The second aim was to characterize the antioxidant activity of the ingested ginger extract.

## 2. Materials and Methods

### 2.1. Ethical Consideration

This clinical trial was approved by the Egas Moniz School of Health and Science Ethics Committee (Project Code 519, approval on 23 November 2016). The participation was voluntary and informed consent was obtained from all participants after receiving oral and written information about the study. Data confidentiality and anonymity were guaranteed through a codification attributed to each participant. The experimental procedure involving humans was carried out according to the Declaration of Helsinki and CONSORT guidelines. This clinical trial is registered on Clinicaltrials.gov (NCT05152745).

### 2.2. Participants and Study Design

This randomized controlled clinical trial, blind to the researcher who performed the statistical analysis, was conducted at Campus Universitário Egas Moniz, Monte de Caparica, Portugal. Twenty-four nondiabetic male and female participants between ages 18 and 40 years were selected. After eligibility criteria were confirmed, participants were sequentially numbered and randomly placed in an intervention group (n = 12) or a control group (n = 12). 

The eligibility and inclusion criteria included subjects of both genders, without glucose metabolism alteration (fasting blood glucose < 126 mg/dL or 6.99 mmol/L). Exclusion criteria included subjects who fasted less than 8 or more than 10 h, were under medication for glycemia control, had gastrointestinal symptoms or disease, pregnant or lactating women, and subjects with an allergy to ginger. Participants were asked not to ingest ginger on the day before the intervention. 

After 8 h fasting, the intervention group performed an oral glucose tolerance test (OGTT), immediately followed by ginger extract administration; the control group performed an OGTT administration alone.

### 2.3. Ginger Extract Preparation

The ginger powder (*Zingibre officinalle* Roscoe) was obtained from a Portuguese company of Indian origin (batch number LI1GIGRNT150012) and stored under standard environmental conditions (21–23 °C, 50–60% humidity) until needed. Ginger powder was individually weighed (0.2 g each dose) and added to 100 mL water, thus producing the ginger aqueous extract, which was boiled for 10 min. After cooling at room temperature, the ginger extract solution was distributed to each participant. This method was adapted from Wilkinson, J. M. (2000) [24]. The ginger extract obtained was subject to total phenolic and flavonoid content determination, as well as radical inhibition assay.

### 2.4. Intervention

Blood samples were collected from each participant after overnight fasting (8 h), using capillary drop blood, before the intervention (t0). The control group ingested an oral glucose solution (75 g of dextrose in 200 mL water) [25] and the intervention group ingested a ginger aqueous extract solution immediately after the oral glucose solution (75 g of dextrose in 200 mL water). Blood samples were collected at 30, 60, 90, and 120 min after glucose solution and/or ginger extract ingestion in both groups. The blood glucose level analysis was performed using a strip for a glucose meter (Onetouch Select Plus Flex), a sterilized lancet, and glucose meter equipment. 

### 2.5. Data Collection

General characteristics of the participants were collected through a questionnaire, including age and anthropometric parameters (weight, height, and body mass index). A 24 h dietary recall questionnaire was administered to participants the day before the intervention. The 24 h recall was instructed by an investigator to complete the food record. The ingested food quantity was estimated using a picture book. The Food Processor SQL (version 10.5.0) was used in order to obtain total energy (Kcal), total carbohydrates (g), total protein (g), and total lipid (g) mean intake.

### 2.6. Chemical Analysis

Folin–Ciocalteu and gallic acid-1-hydrate (C_6_H_2_(OH)_3_COOH·H_2_O) were from PanReac (Cascais, Portugal). Quercetin dihydrate (C_15_H_10_O_7_·2H_2_O) was from Extrasynthese (Lyon, France). Anhydrous aluminum chloride, potassium acetate, sodium carbonate, and Tris(hydroxymethyl)amino methane were from Merck (Alges, Portugal). Phenazine methosulfate (PMS), nicotinamide-adenine dinucleotide hydride (NADH), and nitro-blue tetrazolium chloride (NBT) were from Sigma Aldrich (Lisbon, Portugal). All reagents were pro-analysis grade. All absorbance measurements were performed in a Perkin–Elmer (Lisbon, Portugal) Lambda 25. The reagents were weighed on an analytical balance (Sartorius, ±0.00001 g) (Lisbon, Portugal). 

### 2.7. Total Phenolic Content Determination

The total phenolic content quantification of 7 ginger extract samples was determined according to the Folin–Ciocalteu method [26]. The total phenolic content was expressed as mg gallic acid equivalent (GAE)/L of ginger extract. 

### 2.8. Flavonoid Content Determination

The total flavonoid content quantification of 7 ginger extract samples was determined according to the Prabha method [26]. The total flavonoid content was expressed as mg quercetin equivalent (QCE)/L of ginger extract.

### 2.9. Radical Inhibition Assay

The superoxide anion (O_2_^∙−^) scavenging activity of the ginger extract was determined based on the Morais and Alam methods [27,28]. The superoxide anion was generated by reacting phenazine methosulfate (PMS), nicotinamide adenine dinucleotide hydride (NADH), and oxygen, causing a reduction of NBT in Formazan. A volume of 0.5 mL of ginger extract was added to 2 mL of a solution containing NADH (189 μM) and nitroblue tetrazolium (NBT) (120 μM) with Tris-HCl (40 mM, pH = 8). The reaction started after the addition of 0.5 mL of PMS (60 μM). After 5 min of incubation, control absorbance was measured at 560 nm at room temperature. The percentage of superoxide anion inhibition capacity was calculated using the following equation:

$$Inhibition\;capacity\;\left(\%\right)=\frac{Absorvance\;\left(control\right)-Absorvance\left(sample\right)}{Absorvance\;\left(control\right)}\times 100$$

### 2.10. Statistical Analysis

Statistical analysis of the data was performed using SPSS^®^ (Statistical Package for Social Sciences), version 25.0 software. Descriptive statistics data are reported as the mean ± SD (standard deviation) or SEM (standard error of the mean). Repeated measures of ANOVA of mixed type were used to assess the difference between the 2 groups for postprandial blood glucose at different times. After assumption verification, differences between the 2 groups for total energy, total carbohydrates, total protein and total lipid intake, maximum concentration (Cmax), variation of maximum concentration (ΔCmax), and incremental area under the curve (AUCi) of glucose were assessed using the independent samples *t*-test. The AUCi was calculated using GraphPad Prim (version 7.03) software. All statistical tests were performed at the 5% level of significance.

The sample size required for the study was calculated by simulation using G-Power Software version 3.1.9.4 with a statistical significance of 5% for an expected medium to a large effect size of 20%. Additionally, a low correlation (0.40) was assumed among repeated measures and a sphericity correction epsilon of 0.65.

## 3. Results

### 3.1. Participant Enrollment

**Figure 1:**
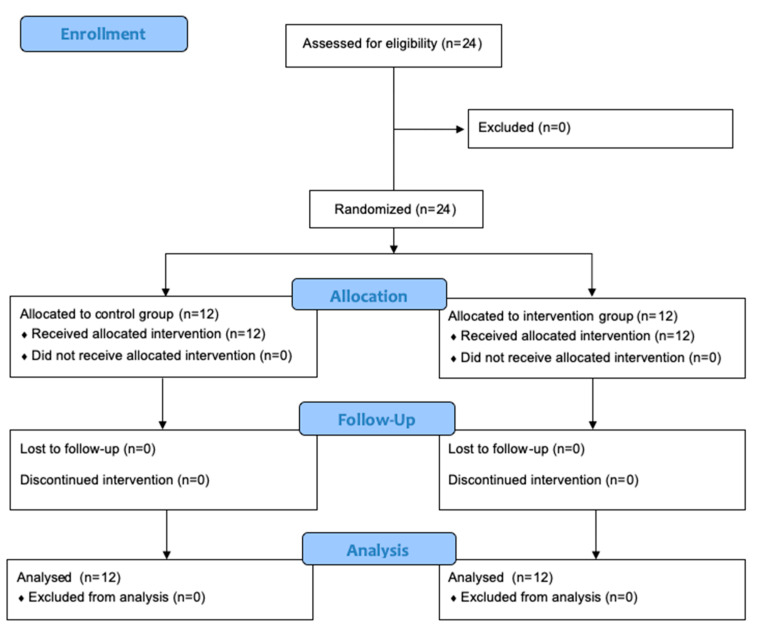
CONSORT flow diagram of study.

In accordance with the CONSORT participant sample description, a total of twenty-four participants were enrolled in and completed the study, twelve for each group, as shown in Figure 1.

### 3.2. Participant Characteristics

The general characteristics of nondiabetic male and female participants are shown in Table 1. A total of 24 participants, 12 subjects in the intervention group (four male, eight female) and 12 subjects in the control group (five male, seven female), completed the study. Participants from both groups did not significantly differ in age (*p* = 0.173), body mass index (*p* = 0.116), weight (*p* = 0.725), or height (*p* = 0.386).

The total nutritional composition of meals at the day before the intervention was analyzed in each participant of both groups. Non-significant differences (*p* > 0.05) were observed in carbohydrates and lipids between groups, as shown in Table 2. The total protein mean and total energy intake was significantly higher in the intervention group compared to the control group (*p* < 0.05).

### 3.3. Glycemic Response

Blood glucose levels were measured during an oral glucose tolerance test (OGTT) in the control and intervention groups, as shown in Table 3. The repeated measures ANOVA of mixed type showed that there was a significant interaction between the independent and the repeated measures factors (*p* < 0.001), which means that there are differences in postprandial blood glucose levels between groups, depending on the moment of measurement. Additionally, the differences in blood glucose levels between different measurement times change depending on the group.

The intervention group showed a significantly decreased blood glucose incremental area under the curve (*p <* 0.001) and variation of blood glucose maximum concentration (*p <* 0.001) compared to the control group (Table 4).

### 3.4. Total Phenols, Flavonoid, and Antioxidant Activity 

The total phenol and flavonoid contents of the ginger extract used in this study are shown in Table 5. The results revealed a high total phenol (13.85 ± 0.1 mg GAE/L extract) and flavonoid (3.35 ± 0.2 mg QCE/L extract) content. 

Additionally, the ginger extract showed a high inhibitory capacity for superoxide radical scavenging (45.73%) and an IC50 of 15.66 mgGAE/L.

## 4. Discussion

The main aim of our study was to investigate if ginger extract improved the postprandial glucose concentration in nondiabetic adults. The findings of our study revealed that the ingestion of ginger aqueous extract (0.2 g/100 mL) improved the glycemic response in nondiabetic subjects compared to the control group. Data analysis showed a significant interaction between the independent and repeated measures factors (*p* < 0.001), which means that there are differences in postprandial blood glucose mean values between groups, depending on the moment of measurement. In addition, the results showed that the postprandial glycemia between different moments changed depending on the group.

The ginger extract reduced the blood glucose incremental area under the curve (AUCi) in the intervention group (169.75 ± 17.3) compared to the control group (334.43 ± 32.4) (*p* < 0.001), and the glucose maximum concentration in the intervention group (7.72 ± 0.28 mmol/L) compared to the control group (9.57 ± 0.43) (*p* < 0.001). These results may be associated with the potential properties of ginger’s bioactive compounds, namely the insulin-mimetic action, leading to increased glucose uptake through the upregulation of GLUT4 expression [16]. 

Furthermore, the results obtained from the postprandial glycemic response during the oral glucose tolerance test allow us to conclude that they are different between groups and suggest a beneficial effect on the postprandial glycemic response after ingestion of ginger extract. According to the literature, the glycemic response depends on the nutritional macronutrient composition of the meals [29]. In fact, in the present study, the average total protein intake on the day before the intervention (89.86 ± 10.63). in the intervention group was significantly (*p* = 0.011) higher than the control group (56.33 ± 4.85). The effect of protein intake on blood glucose has been studied in the literature using different methodological approaches. Khan et al. (1992) showed that the ingestion of 50 g of protein in the form of cottage cheese did not significantly reduce plasma glucose concentration compared with the control group (water alone) for 8 h [27]. In addition, Khoury et al. (2010) demonstrated that postprandial glucose peaks were significantly lower following a high-protein meal, compared with a high-carbohydrate meal [29]. Different studies have also evaluated the effect of protein ingestion in glycemic response through blood glucose concentration analysis for 180 min post-meal. The whey protein and milk protein co-ingestion with mixed meals improves postprandial glycemia [28,30]. On the other hand, in Paterson M. et al.’s study, dietary protein does not seem to influence glycemic control in nondiabetic individuals [31]. In this context, due to the diversity of methods and results in the literature, the influence of protein intake on postprandial glucose is not fully understood. For this reason, although the results showed a beneficial effect of ginger extract ingestion on glycemia, further studies with homogeneous and comparable sample sizes, methodologies, and dietary patterns should be employed.

According to the literature, not many clinical trials have investigated the effect of ginger extract on postprandial glycemia. Most studies evaluate the effect of ginger on fasting glycaemia in diabetic patients. Additionally, the findings regarding ginger’s effect on glucose homeostasis seem to be contradictory. A recent meta-analysis that included eight randomized trials, with a total of 454 type 2 diabetic participants, revealed that ginger ingestion did not significantly improve glycaemia response in patients with type 2 diabetes mellitus (*p* = 0.16). Additionally, this study also showed that HbA1c significantly improved in the participants with ginger ingestion (*p* = 0.02) from the baseline to the follow-up, suggesting that ginger may have a beneficial impact on glucose control over a longer period of time [20]. 

Other studies have reported that ginger powder significantly reduces fasting glucose concentration. In a double-blind placebo-controlled randomized clinical trial, type 2 diabetic patients revealed significant differences in serum glucose (*p* < 0.001) in the intervention group compared with the control group after 3 months of the intervention (3 g per day of powdered ginger) [32]. Additionally, in Arablou et al.’s study, the ingestion of 1.6 mg powdered ginger (capsule) per day for 12 weeks significantly lowered (*p* = 0.02) fasting plasma glucose, compared with the placebo group [33]. The ingestion of 2 g of ginger supplement for 12 weeks in type 2 diabetic patients also reduced the concentration of serum blood glucose (*p =* 0.000) [34]. In addition to this beneficial effect on glycemia, ginger powder has been shown to decrease serum insulin resistance [35] and significantly improve insulin levels and hemoglobin A1c [33]. In a randomized double-blind placebo-controlled trial with 64 type 2 diabetic patients (28 patients in the ginger group; 30 patients in the placebo group), the ginger supplementation in lower doses (2 g/day) for 2 months had a beneficial effect on insulin levels, but no significant change on fasting blood glucose. The dietary intakes of the participants revealed no significant difference in macronutrient intake between groups, both at the baseline and at the end of the study [36]. 

The discrepancy in the literature results could be attributed to heterogenicity of the study designs, ginger chemical composition, doses, formulations, extraction processes, and population samples [37]. Nevertheless, according to recent data, the consumption of ginger seems safe and acts beneficially on human health and well-being, highlighting the potential effect the glycemic control [38].

The mechanism of action of ginger extract responsible for glucose homeostasis control effects can be supported by animal and in vitro studies. The administration of 200 mg/kg of gingerol for 4 weeks significantly potentiates GLP-1-mediated glucose-stimulated insulin-secretion pathway in pancreatic beta cells of treated type 2 diabetic mice, compared to untreated type 2 diabetic mice [16]. The increase in insulin secretion through endocrine hormones can be related to a beneficial effect on plasma glucose concentration regulation. In C2C12 cells, the polyphenol-rich Indian ginger extract increased insulin-stimulated glucose uptake [39]. Moreover, different studies explored several underlying mechanisms promoted by different ginger bioactive compounds, which can play a role in glucose control in peripheral tissues. The [6]-Gingerol increased the glucose-stimulated insulin secretion [16]. This compound upregulated and activated cAMP, PKA, and CREB in the pancreatic islets, which can contribute to the insulin-secretion pathway [16]. In addition, [6]-Gingerol regulated the Rab27a GTPase in pancreatic islets, leading to the exocytosis of insulin-containing dense-core granules [16]. Additionally, S-[8]-gingerol seems to increase the protein level of GLUT 4 in a dose-dependent manner in L6 myotubes [40]. 

Moreover, our study confirms that ginger aqueous extract possesses a high-antioxidant activity through the free radical scavenging capacity. This finding could be correlated with a high-polyphenolic content observed in ginger extract since, according to the literature, there is a significant correlation between free radical scavenging capacity and total phenolic content [41].

According to Manjunathan et al., the antioxidant properties and phenolic content of ginger aqueous extract could also be attributed to gingerol bioactive compound activity [42]. These findings are in accordance with the Fathi study, in which hydroethanolic extract of ginger demonstrated a good level of DPPH scavenging activity and total phenolic content per gram of dry extract [43]. The bioactive compounds identified in ginger, namely [6]-gingerol, [8]-gingerol, [10]-gingerol, and [6]-shogaol showed important scavenging activities with IC50 of 26.3, 19.47, 10.47, and 8.05 µM against DPPH radical and with IC50 of 4.05, 2.5, 1.68, and 0.85 µM against superoxide radical, respectively [44]. Since hyperglycemia induces free radical formation, including the superoxide anion [4], the administration of ginger extract may also contribute beneficially to oxidative damage prevention through its high inhibitory capacity for superoxide radical scavenging (45.73%).

Limitations of this study include the unblinded design regarding investigators and the study participants, which was not possible given the nature of the study. The authors did not evaluate the plasma insulin concentration and plasma glucagon-like peptide (GLP-1), which are important in analyzing the effect of ginger extract on GLP-1 and insulin secretion, allowing us to understand its mechanism of action. Additionally, it would be interesting to test other ginger aqueous extract doses in order to explore the eventual postprandial glycemia ginger extract dose-dependence. Further research should be undertaken with a larger sample size and performed over a longer period as part of a mixed-meal daily intake, in order to verify the effect of ginger extract in the long term.

## 5. Conclusions

The current study indicates that the ingestion of ginger (*Zingiber officinale* Roscoe) aqueous extract (0.2 g/100 mL) reduces blood glucose incremental area under the curve and postprandial maximum glucose level variation in nondiabetic subjects. In addition, ginger extract possesses substantial antioxidant activity through free radical scavenging activity. The present study contributes to the support of the beneficial properties of ginger (*Zingiber officinale* Roscoe), suggesting that this herb extract may be effective against hyperglycemic status in nondiabetic subjects.

## Figures and Tables

**Table 1:** Baseline characteristics of the study participants (n = 12, each group).

Parameters	Control Group Mean ± SEM	Intervention Mean ± SEM	*p*-Value ^1^
Age (years)	26.92 ± 1.64	30.50 ± 1.94	0.173
Body mass index (Km/m^2^)	22.74 ± 0.48	23.87 ± 0.50	0.116
Weight (Kg)	64.09 ± 2.88	65.46 ± 2.53	0.725
Height (m)	1.68 ± 0.03	1.64 ± 0.02	0.386

^1^ *p*-Value was calculated by independent samples *t*-test.

**Table 2:** Mean values of total protein (g), carbohydrate (g), lipid (g), and energy intake on the day before intervention in control and intervention groups (n = 12, each group).

Nutritional Parameters	Control Group Mean ± SEM	Intervention Mean ± SEM	*p*-Value ^1^
Protein (g)	56.33 ± 4.85	89.86 ± 10.63	0.011
Carbohydrate (g)	247.81 ± 21.91	306.79 ± 22.73	0.075
Lipid (g)	73.93 ± 6.56	79.71 ± 8.99	0.608
Total energy intake (Kcal)	1826.18 ± 110.35	2283.04 ± 179.09	0.041

^1^ *p*-Value was calculated by independent samples *t*-test.

**Table 3:** Blood glucose levels (mmol/L) obtained for control (n = 12) and intervention (n = 12) groups at different time point: before intervention (t0), and after 30 (t30), 60 (t60), 90 (t90), and 120 (t120) minutes after intervention.

Time Point	Control Group Mean ± SD	Intervention Mean ± SD
t0	4.82 ± 0.12	4.93 ± 0.74
t30	9.57 ± 0.43	7.72 ± 0.28
t60	8.76 ± 0.66	6.56 ± 0.21
t90	6.64 ± 0.36	5.55 ± 0.19
t120	6.03 ± 0.28	5.55 ± 0.18

**Table 4:** Blood glucose incremental area under the curve (AUCi), blood glucose maximum concentration (Cmax), and variation of blood glucose maximum concentration (∆Cmax) mean values in control and intervention groups (n = 12, each group).

Clinical Parameters	Control Group Mean ± SEM	Intervention Mean ± SEM	*p*-Value ^1^
AUCi (t0–t120 min)	334.43 ± 32.40	169.75 ± 17.30	<0.001
Cmax (mmol/L)	9.57 ± 0.43	7.72 ± 0.28	<0.001
∆Cmax (mmol/L	4.75 ± 0.46	2.80 ± 0.26	<0.001

^1^ *p*-Value was calculated by independent samples *t*-test. Abbreviations: AUCi (incremental area under the curve); Cmax (maximum concentration); ∆Cmax (variation of maximum concentration).

**Table 5:** Total phenol and flavonoid content mean values of ginger extract (n = 7).

Compounds	Mean ± SEM
Total phenols (mg GAE/L) y = 6.431 × 10^3^x + 1.79 × 10^−2^ (R^2^ = 0.9992)	13.85 ± 0.15
Total flavonoids (mg QCE/L) y = 2.5015 × 10^−2^x − 1.2673 × 10^−2^ (R^2^ = 0.99939)	3.35 ± 0.16

GAE—gallic acid equivalent; QCE—quercetin equivalent.

## Data Availability

The data presented in this study are available on request from the last author.
